# Bibliometric analysis of leishmaniasis research in Medline (1945-2010)

**DOI:** 10.1186/1756-3305-6-55

**Published:** 2013-03-07

**Authors:** José M Ramos, Gregorio González-Alcaide, Máxima Bolaños-Pizarro

**Affiliations:** 1 Department of Internal Medicine, Hospital General Universitario de Alicante, Alicante, Spain; 2 Department of Medicine, Universidad Miguel Hernández de Elche, Alicante, Spain; 3 Department of History of Science and Documentation, Universitat de València, Facultad de Medicina y Odontología, Valencia, Spain; 4 Universitat de València, Valencia, Spain

**Keywords:** Leishmaniasis, *Leishmania*, Bibliometry, Scientific production, Mapping, Leishmaniasis visceral, Leishmaniasis cutaneous, Leishmaniasis mucocutaneous, Diffuse cutaneous Leishmaniasis

## Abstract

**Background:**

Publications are often used as a measure of success of research work. Leishmaniasis is considered endemic in 98 countries, most of which are developing. This article describes a bibliometric review of the literature on leishmaniasis research indexed in PubMed during a 66-year period.

**Methods:**

Medline was used via the PubMed online service of the US National Library of Medicine. The search strategy was *Leishmania* [MeSH] or leishmaniasis [MeSH] from 1 January 1945 until 31 December 2010. Neither language nor document type restrictions were employed.

**Results:**

A total of 20,780 references were retrieved. The number of publications increased steadily over time, with 3,380 publications from 1945-1980 to 8,267 from 2001-2010. Leishmaniasis documents were published in 1,846 scientific journals, and *Transactions of the Royal Society of Tropical Medicine and Hygiene* (4.9%) was the top one. The USA was the predominant country by considering the first author’s institutional address (16.8%), followed by Brazil (14.9%), and then India (9.0%), however Brazil leads the scientific output in 2001-2010 period (18.5%), followed by the USA (13.5%) and India (10%). The production ranking changed when the number of publications was normalised by population (Israel and Switzerland), by gross domestic product (Nepal and Tunisia), and by gross national income per capita (India and Ethiopia). For geographical area, Europe led (31.7%), followed by Latin America (24.5%).

**Conclusions:**

We have found an increase in the number of publications in the field of leishmaniasis. The USA and Brazil led scientific production on leishmaniasis research.

## Background

Leishmaniasis is a group of diseases caused by protozoan parasites of the *Leishmania* genus, order *Kinetoplastida*. More than 20 *Leishmania* species are considered human pathogens. Leishmaniasis occurs on four continents and is considered endemic in 98 countries and three territories, most of which are low- and middle-income [1,2].

Leishmaniasis is a poverty-related disease in which poverty and disease reinforce each other in a vicious cycle. Poverty determinants like malnutrition, displacement, poor housing, illiteracy, gender discrimination, immune system weakness, and lack of resources have been reviewed elsewhere [3]. Leishmaniasis is still one of the world’s most neglected diseases; 350 million people are considered at risk of contracting leishmaniasis, and some 1 million new cases occur yearly [2]. In the past 10 years, there have been major scientific breakthroughs in the treatment, diagnosis and prevention of leishmaniasis, and the cost of several key medicines has been reduced [4,5].

Research is important in a country’s development and progress. Biomedical research projects usually lead to publications in the serial literature. Original articles allow investigators to present their scientific observations, and the publication of an investigator’s project allows the information to be shared by the scientific community. Publications are often used as a measure of success of research work. In recent years, there has been growing interest in developing scientific indicators capable of facilitating the analysis of the results of research activities [6].

The term “neglected tropical diseases”, which was coined in the mid 1990s, has become a “brand-name” referring to a group of diseases which are especially endemic in low-income populations living in tropical and subtropical countries. Since then, there has been a growing interest in research and there are specific journals now available for getting this research published in the peer-reviewed literature [7]. There are international bibliometric studies in different fields of medicine [8-10] and/or tropical medicine [11-14]. There have been publications analyzing the research production in other neglected tropical diseases (NTD), such as schistosomiasis [15,16], leprosy [16,17], and Chagas disease [16,18]. As for leishmaniasis, one quantitative study analysing literature research output for the period between 1957 and 2006 using the Web of Science has been published [19]. There are other studies analyzing the scientific production and productivity of Iranian institutes in the field of leishmaniasis using the Medline database [20]. The PubMed database offers the possibility of analysing Medical Subject Headings (MeSH), MeSH categories articles and more biomedical journals than the Web of Science. Although the previous publications allowed a bibliometric analysis of research output on leishmaniasis, mapping leishmaniasis research to other aspects such as authorship and clinical forms of leishmaniasis is still pending. The aim of this study was to investigate leishmaniasis research output using PubMed over a period of 66 years (1945-2010) by journal of publication; animal or human MeSH, taking advantage of the fact that in PubMed you can filter the search results by considering human or animal studies; forms of the disease and author production in terms of number of publications per author.

## Methods

The Medline database, accessible free of charge through the PubMed platform, was selected as the most suitable for references to leishmaniasis publications due to its volume and coverage. Furthermore, it uses a controlled vocabulary, the Medical Subject Headings (MeSH) thesaurus, a hierarchical structure made up of 26,000 descriptors and over 213,000 entry terms, which allowed us to perform accurate searches. This database is easily accessible and widely used [8,9,21]. PubMed (http://www.ncbi.nlm.nih.gov/pubmed) was accessed online on 10 February 2012. The subject content analysis of records was conducted according to the MeSH structure. For retrieving documents, a search was composed with the MeSH terms or descriptors ‘*leishmaniasis’* or ‘*Leishmania’*. The study period was from 1 January 1945 to 31 December 2010, grouped by 5-year increments because PubMed citations go back to 1945. We did not consider any language or document type restriction in the search, in order to analyse publication patterns of all publications on Leishmaniasis.

The *document type* used in our study refers to the type of article and its financing. The impact factor of a journal and its ranking was obtained from the *Journal Citation Report* (JCR) 2010 Science Edition [22].

The productivity by country was analysed considering the number of papers and the percentage of world production. The institutional affiliation is only included for the first participating author since 1986 in the PubMed database in *Journal articles* and *Review*. Indicators of each country’s productivity between 2001-2010 period were standardised with respect to the population, gross domestic product (GDP), gross national income (GNI) per capita and the health expenditure per capita. To calculate the publications per million inhabitants (population index), per billion of GDP (US dollars) (GDP index), per 100 US dollars of GNI per capita (GNI per capita index), and per 10 US dollars of health expenditure (HE) per capita (HE per capita index), data were obtained from *Word Development Indicators* from the online databases of the World Bank [23].

Based on geographic, scientific and economic criteria, the world was divided into seven regions: i) Europe; ii) North America (United States of America [USA] and Canada), iii) Latin America and the Caribbean; iv) North Africa and the Middle East (including Turkey); v) Africa; vi) Asia; and vii) Oceania. According to the MeSH term, the forms of diseases were the following: ‘*Leishmaniasis, visceral’*, ‘*Leishmaniasis, cutaneous’*, ‘*Leishmaniasis mucocutaneous’*, and ‘*Leishmaniasis diffuse cutaneous*’. ‘*Leishmaniasis, cutaneous’* and ‘*Leishmaniasis, diffuse cutaneous’* have been included in PubMed since 1992.

The information obtained from the registers was introduced into a database using Microsoft Access 2007. A standardisation process was carried out to consolidate variations in author names. The criterion followed in this process was the occurrence of the institutional signature associated with the variations in names and surnames. Research output was analysed by country, geographic area, and forms of leishmaniasis. Publication authorship was analysed by the forms of leishmaniasis.

## Results

**Figure 1:**
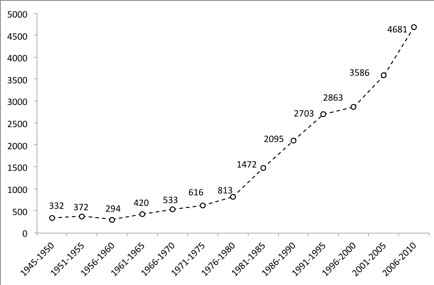
Number of leishmaniasis disease research publications in PubMed (○) between 1945 and 2010 per 5-year period.

In the PubMed database, 20,780 references were retrieved for the entire study period. There were 3,380 (16.3%) publications from 1945 to 1980, 3,567 (17.2%) from 1981 to 1989, 5,566 (26.8%) from 1991 to 2000, and 8,267 from 2001 to 2010 (39.8%). Figure 1 shows the numbers of PubMed publications on leishmaniasis research during the 66-year study period in five-year periods. The five-year average increase of publications was +10.5% throughout the study period, although this percentage was much higher from 1981 to 1985 (+81.1%), 1961 to 1965 (+42.9%), and from 1986 to 1990 (+42.3%). This percentage was less from 1956 to 1960 (-21.0%) and 1996 to 2000 (+5.9%). After fitting the number of publications over time, a better fit was observed for a straight line (coefficient of determination for linear fit, R^2^ = 0.91) than for an exponential curve (R^2^ = 0.81).

### Language of publication

The primary language was English (82.8%), followed by French (4.2%), Portuguese (2.8%), Spanish (2.6%), Russian (2.5%), German (1.3%), Italian (0.8%), Chinese (0.5%), undetermined (1.8%) and others (0.8%).

### Journal of publication

The 20,700 retrieved articles were published in 1,846 scientific journals. Eight journals accounted for 21.4% of the leishmaniasis journal literature. About one-half of the literature is concentrated in 50 journals, while the remaining half is scattered throughout 1,796 journals. Moreover, 743 journals published only one paper on leishmaniasis. Table 1 shows a list of the 50 journals with the highest number of papers published from 1945-2010, as well as their impact factors for the year 2010, subject category according to the JCR classification and language. Seven of these journals were not included in JCR because they did not have an impact factor, and three of these journals were not published in 2010. The remaining journals were included in at least one of 22 subject categories. *Transactions of the Royal Society of Tropical Medicine and Hygiene* leads the number of leishmaniasis documents published during 1945-2010 period (n = 1,018). The source journals mainly include the fields of parasitology (n = 13), immunology (n = 11), tropical medicine (n = 9), biochemistry and molecular biology (n = 5) and microbiology (n = 5), public health (n = 5), among others.

**Table 1:** The 50 journals with the highest number of leishmaniasis articles published during the 1945-2010 period

**Journal**	**N. of docs**	**%**	**Impact factor 2010**	**Journal category (ranking)**	**Language**
*Transactions of the Royal Society of TropicalMedicine and Hygiene*	1.018	4.9	2.832	Public. Environmental & Occupational Health (30 of 142)	Eng
				Tropical Medicine (4 of 19)	
*The American Journal of Tropical Medicine and Hygiene*	679	3.3	2.446	Public. Environmental & Occupational Health (38 of 142)	Eng
				Tropical Medicine (5 of 19)	
*Molecular and Biochemical Parasitology*	599	2.9	2.875	Biochemistry & Molecular Biology (189 of 236)	Eng
				Parasitology (6 of 32)	
*Memórias do Instituto Oswaldo Cruz*	438	2.1	2.058	Parasitology (13 of 32)	Eng
				Tropical Medicine (7 of 19)	
*Experimental Parasitology*	428	2.1	1.869	Parasitology (14 of 32)	Multi
*Journal of immunology (Baltimore. Md. : 1950)*	427	2.1	5.747	Immunology (20 of 134)	Eng
*Annals of Tropical Medicine and Parasitology*	426	2.1	1.579	Parasitology (20 of 32)	Eng
				Public. Environmental & Occupational Health (86 of 142)	
				Tropical Medicine (8 of 19)	
*Infection and Immunity*	423	2.0	4.098	Immunology (33 of 134)	Eng
				Infectious Diseases (11 of 58)	
*Meditsinskaia Parazitologiia i Parazitarnye Bolezni*	352	1.7	NI	-	Rus
*The Journal of Biological Chemistry*	295	1.4	5.328	Biochemistry & Molecular Biology (50 of 286)	Eng
*Acta Tropica*	291	1.4	2.262	Parasitology (10 of 32)	Eng
				Tropical Medicine (6 of 19)	
*Revista da Sociedade Brasileira de Medicina Tropical*	277	1.3	0.580	Tropical Medicine (14 of 19)	Mul
*Parasitology Research*	216	1.0	1.812	Parasitology (15 of 32)	Eng
*Antimicrobial Agents and Chemotherapy*	211	1.0	4.672	Microbiology (18 of 107)	Eng
				Pharmacology & Pharmacy (26 of 252)	
*The Journal of Parasitology*	203	1.0	1.208	Parasitology (21 of 32)	Eng
*Parasitology*	201	1.0	2.522	Parasitology (7 of 32)	Eng
*International Journal of Dermatology*	195	0.9	1.265	Dermatology (36 of 55)	Eng
*The Journal of Infectious Diseases*	188	0.9	6.288	Immunology (19 of 134)	Eng
				Infectious Diseases (5 of 58)	
				Microbiology (12 of 112)	
*Veterinary Parasitology*	187	0.9	2.331	Parasitology (9 of 32)	Eng
				Veterinary Sciences (9 to 145)	
*Journal of the Egyptian Society of Parasitology*	181	0.9	NI	-	Eng
*Revista do Instituto de Medicina Tropical de São Paulo*	176	0.8	0.934	Tropical Medicine (12 of 19)	Multi
*European Journal of Immunology*	166	0.8	4.942	Immunology (22 of 134)	Eng
*Vaccine*	152	0.7	3.572	Immunology (43 of 134)	Eng
				Medicine. Research & Experimental (25 of 106)	
*International Journal for Parasitology*	149	0.7	3.822	Parasitology (4 of 32)	Eng
*Clinical Infectious Diseases*	142	0.7	8.186	Immunology (11 of 134)	Eng
				Infectious Diseases (2 of 58)	
				Microbiology (9 of 112)	
*Parasite Immunology*	138	0.7	2.328	Immunology (8 of 134)	Eng
				Parasitology (8 of 32)	
*Proceedings of the National Academy of Sciences of the United States of America*	129	0.6	9.971	Multidisciplinary Sciences (3 of 59)	Eng
*The Journal of Experimental Medicine*	126	0.6	14.776	Immunology (2 of 134)	Eng
				Medicine. Research & Experimental (5 of 106)	
*Clinical and Experimental Immunology*	122	0.6	3.134	Immunology (56 of 134)	Eng
*Bulletin de la Société de Pathologie Exotique et de ses Filiales (a)*	122	0.6	NI	-	Fre
*Lancet*	121	0.6	33.633	Medicine. General & Internal (2 of 153)	Eng
*Journal of Clinical Microbiology*	116	0.6	4.220	Microbiology (20 of 112)	Eng
*Indian Journal of Medical Research*	112	0.5	1.826	Immunology (106 of 134)	Eng
				Medicine. General & Internal (44 of 153)	
				Medicine. Research & Experimental (56 of 106)	
*Tropical Medicine & International Health*	109	0.5	2.967	Public. Environmental & Occupational Health (29 to 142)	Eng
				Tropical Medicine (3 of 19)	
*The Journal of Protozoology (3)QUÉ ES ESTO*	105	0.5	NI	-	Eng
*East African Medical Journal*	95	0.5	NI	-	Eng
*Journal of Medical Entomology*	94	0.5	1.925	Entomology (12 of 83)	Eng
				Veterinary Sciences (15 of 145)	
*Parassitologia*	91	0.4	NI	-	Ita
*Nucleic Acids Research*	90	0.4	3.836	Biochemistry & Molecular Biology (30 of 286)	Eng
*Journal of Communicable Diseases*	84	0.4	NI	-	Eng
*Annales de Parasitologie Humaine et Comparée (c)*	83	0.4	NI	-	Fre
*Trends in Parasitology*	82	0.4	4.906	Parasitology (2 of 32)	Eng
*Microbes and Infection*	82	0.4	2.726	Immunology (70 of 134)	Eng
				Microbiology (42 of 107)	
				Virology (16 of 33)	
*Cadernos de Saúde Pública*	80	0.4	0.987	Public. Environmental & Occupational Health (107 *of* 142)	Por
*PLoS Neglected Tropical Diseases*	80	0.4	4.752	Parasitology (3 of 32)	Eng
				Tropical Medicine (1 of 19)	
*Journal of Medicinal Chemistry*	79	0.4	5.527	Chemistry. Medicinal (3 of 54)	Eng
*Bioorganic & Medicinal Chemistry*	76	0.4	2.906	Biochemistry & Molecular Biology (134 of 286)	Eng
				Chemistry. Medicinal (15 of 54)	
				Chemistry. Organic (16 of 56)	
*The Biochemical Journal*	75	0.4	5.016	Biochemistry & Molecular Biology (134 of 286)	Eng
*Médecine Tropicale : Revue du Corps de Santé Colonial*	75	0.4	NI	-	Fre
*Science (New York. N.Y.)*	75	0.4	31.377	Multidisciplinary Sciences (2 of 59)	Eng

Their impact factors for the year 2010. Journal category with ranking from the *Journal Citation Report* and language of publication.

NI: Not included in 2010 JCR Science Edition. Docs: documents.

*Eng*, English; *Fre*, French; *Ita*, Italian; *Multi*, Multi-language; *Por*, Portuguese; *Spa*, Spanish; *Rus*, Russian.

(a) Publication end year 1989.

(b) Publication end year 1992.

(c) Publication end year 1993.

### MeSH

**Figure 2:**
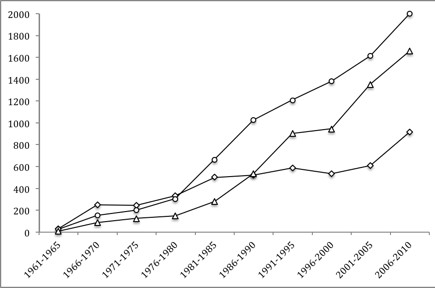
**Number of leishmaniasis disease research publications in PubMed with *Animals *(○), *Animals & Humans *(**∆**) and *Humans *(◊) MeSH, between 1945 and 2010 per 5-year period.**

The 30 most frequent MeSH words in documents published during the 1945-2010 period about leishmaniasis are shown in Table 2. *Animals* (41.2%) was the predominant MeSH, followed by *Animals and Humans* (29.0%), and *Humans* (21.8%). Figure 2 shows the numbers of PubMed publications on leishmaniasis research with MeSH *Animals*, *Animals and Humans*, and *Humans* during the 66-year study period by five-year periods. After fitting the number of publications over time, a better fit was observed for a straight line (R^2^ = 0.98) for *Animals*; for *Animals and Humans*, a better fit was observed for an exponential curve (R^2^ = 0.97) than for a straight line (R^2^ = 0.91); and for *Humans*, a better fit was observed for a line (R ^2^ =0.90) than for an exponential curve.

**Table 2:** The 30 top Medical Subject Headings (MeSH) words in leishmaniasis articles published during the 1945-2010 period

**MeSH**	**N. of documents**	**%**
Animals	8.564	41.2
Animals & Humans	6.036	29.0
Leishmaniasis. Visceral	6.216	29.9
Male	5.514	26.5
Female	5.431	26.1
Leishmania	5.034	24.2
Leishmaniasis	4.991	24.0
Humans	4.531	21.8
Mice	3.852	18.5
Leishmaniasis. Cutaneous	3.848	18.5
Adult	3.295	15.9
Leishmania donovani	2.896	13.9
Antiprotozoal Agents	2.604	12.5
Mice. Inbred BALB C	2.125	10.2
Child	2.039	9.8
Leishmania major	1.937	9.3
Adolescent	1.825	8.8
Middle Aged	1.807	8.7
Macrophages	1.575	7.6
Dogs	1.513	7.3
Molecular Sequence Data	1.447	7.0
Antigens. Protozoan	1.447	7.0
Child. Preschool	1.428	6.9
Leishmania infantum	1.402	6.7
Insect Vectors	1.343	6.5
Protozoan Proteins	1.233	5.9
Antibodies. Protozoan	1.182	5.7
Leishmania mexicana	1.182	5.7
Psychodidae	1.126	5.4
Dog Diseases	1.125	5.4
Cricetinae	1.038	5.0

**Figure 3:**
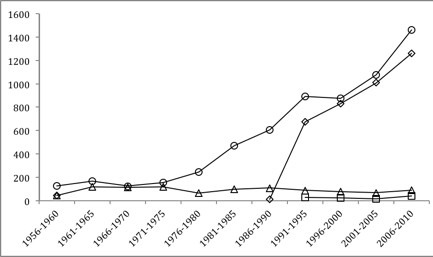
**Number of leishmaniasis disease research publications in PubMed: *visceral leishmaniasis *(○), *mucocutaneous leishmaniasis *(**∆**)**, ***cutaneous leishmaniasis *(◊), and *diffuse cutaneous leishmaniasis *(**+**) MeSH, between 1945 and 2010 per 5-year period.**

The MeSH visceral leishmaniasis, cutaneous leishmaniasis, mucocutaneous leishmaniasis, and diffuse cutaneous leishmaniasis were reported in 29.9%, 18.5%, 4.8% and 0.5%, respectively. Figure 3 shows the numbers of PubMed publications by categories of leishmaniasis during the 66-year study period per five-year period. The main MeSH *Leishmania* species were *L. donovani* (13.9%), *L. major* (9.3%), *L. infantum* (6.7%), *L. mexicana* (5.7%), *L. braziliensis* (3.9%), and *L. tropica* (3.9%).

### Document type of publications

*Journal articles* are the most common document type, accounting for about 86.5% of the total (n = 17,982). *Review* and *Letter* were the second and third most common, with 1,616 (7.8%), and 1,008 (4.9%) documents, respectively. Only 1.1% of the documents were subdivided into *Randomized controlled trials* (n = 234), 0.8% into *Clinical trial* (n = 157), and 0.3% into *Controlled clinical trials* (n = 52). *Case reports* appeared in 2,171 (10.4%) and *Comparative study* in 1,750 (8.4%) documents.

### Publication by country

The first author’s institutional address was available for 13,973 of the 20,780 publication documents (67.2%). One-hundred and seven countries published at least one paper. USA was the predominant country (16.8%), followed by Brazil (14.9%), India (9.0%), the United Kingdom [UK] (7.1%), France (5.8%), Spain (5.3%) and Germany (4.2%). These seven countries contributed 63.1% of all research documents published during the study period (1945-2010) and 30 countries contributed 92.6%. Brazil leads the scientific publications between 2001 and 2010 (18.5%), followed by the USA (13.5%), India (10%), UK (5.8%) and Spain (5.5%).

Table 3 ranks countries in crude numbers of retrieved articles between 2001 and 2010 and numbers corrected by population index, GDP index, GNI per capita index and HE per capita index. When normalised by population, the order of prominence was Israel, Switzerland, Tunisia, Malta and Spain. Normalised by GDP, we found that among low- and middle-income countries, Nepal, Tunisia, Ethiopia, Sudan and Kenya were the most productive. If we calculate the ratio of number of leishmaniasis publications to GNI per capita, India, Brazil, Ethiopia, Nepal and Iran were the most productive. When normalised by HE per capita, the leading order were overpopulated countries: India, Ethiopia, Pakistan, Brazil and Nepal.

**Table 3:** Top 30 countries and world regions ranked according to total number of publications

**Country**	**N. of docs**	**%**	**Country**	Population index*	**Country**	GDP index**	**Country**	GNI per capita index***	**Country**	HE per capita index****
Brazil	1.426	18.47	Israel	16.98	Nepal	4.44	India	96.50	India	227.35
USA	1.040	13.47	Switzerland	14.28	Tunisia	3.51	Brazil	27.08	Ethiopia	39.00
India	773	10.01	Tunisia	11.89	Ethiopia	2.41	Ethiopia	19.12	Pakistan	35.79
UK	450	5.83	Malta	9.88	Sudan	1.59	Nepal	12.88	Brazil	29.52
Spain	427	5.53	Spain	9.78	Kenya	1.37	Iran	9.46	Nepal	22.11
France	376	4.87	Greece	8.63	Brazil	1.29	Pakistan	9.13	Bangladesh	13.33
Germany	302	3.91	Brazil	7.65	Iran	1.25	Sudan	7.39	Iran	13.00
Italy	291	3.77	UK	7.44	Burkina Faso	1.02	Kenya	5.21	Kenya	11.54
Iran	264	3.42	Belgium	7.40	Bolivia	0.83	Bangladesh	3.99	Sudan	11.22
Canada	213	2.76	Portugal	7.32	India	0.80	Colombia	3.77	Tunisia	6.32
Colombia	133	1.72	Canada	6.55	Sri Lanka	0.79	Tunisia	3.73	Colombia	4.94
Tunisia	120	1.55	France	5.94	Colombia	0.79	USA	2.41	Sri Lanka	4.42
Israel	119	1.54	Italy	4.95	Israel	0.77	China	2.09	China	4.22
Turkey	115	1.49	Australia	4.64	Malta	0.63	Egypt	1.94	Egypt	3.78
Venezuela	111	1.44	Czech Republic	4.27	Paraguay	0.59	Turkey	1.73	Turkey	2.77
Switzerland	107	1.39	Netherlands	4.22	Pakistan	0.56	Spain	1.73	Venezuela	2.75
Japan	101	1.31	Venezuela	4.14	Venezuela	0.55	Venezuela	1.69	Burkina Faso	2.22
Australia	96	1.24	Iran	3.76	Afghanistan	0.52	Sri Lanka	1.67	Tanzania	2.11
Greece	96	1.24	Germany	3.67	Cameroon	0.46	Burkina Faso	1.55	Iraq	1.94
Belgium	78	1.01	Sweden	3.63	Albania	0.46	Afghanistan	1.43	Spain	1.92
Portugal	77	1.00	Bahrain	3.51	Panama	0.45	UK	1.23	Cameroon	1.67
Netherlands	69	0.89	USA	3.50	Senegal	0.43	Argentina	1.19	USA	1.56
Argentina	68	0.88	Colombia	3.07	Surinam	0.42	Uganda	1.17	Morocco	1.55
Pakistan	68	0.88	Denmark	2.57	Portugal	0.40	France	1.11	UK	1.48
Mexico	65	0.84	Panama	2.45	Greece	0.39	Tanzania	1.01	Bolivia	1.47
Sudan	55	0.71	Uruguay	2.41	Jordan	0.38	Italy	0.99	Afghanistan	1.43
China	46	0.60	Austria	2.07	Uganda	0.38	Iraq	0.96	Mexico	1.34
Czech Republic	44	0.57	Surinam	2.00	Iraq	0.38	Germany	0.88	Argentina	1.33
Nepal	42	0.54	Cuba	1.96	Spain	0.37	Cameroon	0.87	Uganda	1.29
Ethiopia	39	0.51	Cyprus	1.93	Uruguay	0.36	Mexico	0.84	Yemen	1.28
**World regions**	**N. of docs**	**%**	**World regions**	Population index*	**World regions**	GDP index**	**World regions**	GNI per capita index***	**World regions**	HE per capita index****
Europe	2,452	31.75	Oceania	3.98	Latin America and the Caribbean	0.62	Latin America and the Caribbean	2.14	Latin America and the Caribbean	2.87
Latin America and the Caribbean	1,893	24.51	North America	3.80	North Africa	0.31	North America	1.63	Asia	1.72
North America	1,253	16.23	Latin America and the Caribbean	3.57	Africa	0.30	Asia	0.84	North America	1.21
Asia	1,397	18.09	Europe	3.50	Europe	0.16	Africa	0.74	Africa	1.12
North Africa	459	5.94	North Africa	1.45	Asia	0.13	Europe	0.34	North Africa	0.72
Africa	169	2.19	Asia	0.43	Oceania	0.12	North Africa	0.25	Europe	0.36
Oceania	99	1.28	Africa	0.31	North America	0.09	Oceania	0.18	Oceania	0.18

Publications per inhabitant, per gross domestic product (GDP), per gross national income (GNI) per capita, and health expenditure (HE) per capita in 7.851 leishmaniasis manuscripts with institutional address of the first author (2001-2010). Docs: documents.

* Number of publications per million of population.

** Number of publications per 1 billion US dollars of gross domestic product (GDP).

*** Number of publications per 100 US dollars of GNI per capita.

**** Number of publications per 10 US dollars of HE per capita.

Table 4 ranks countries in crude numbers of retrieved articles, stratified by forms of the leishmaniasis. For visceral leishmaniasis, the main countries were India, Brazil and Spain. Regarding cutaneous leishmaniasis, the countries leading the ranking were Brazil, the USA, and Germany. For mucocutaneous leishmaniasis, Brazil, USA and France led; meanwhile, for diffuse cutaneous leishmaniasis, leading countries were Brazil, Venezuela and USA.

**Table 4:** Top 30 countries and word regions ranked according to total number of publications by forms of leishmaniasis disease

**Visceral leishmaniasis**			**Cutaneous leishmaniasis**			**Mucocutaneous leishmaniasis**			**Diffuse cutaneous leishmaniasis**		
**Country**	**N. of docs**	**%**	**Country**	**N. of docs**	**%**	**Country**	**N. of docs**	**%**	**Country**	**N. of docs**	**%**
India	758	18.6	Brazil	667	19.0	Brazil	133	37.6	Brazil	34	33.3
Brazil	583	14.3	USA	571	16.3	USA	33	9.3	Venezuela	13	12.7
Spain	339	8.3	Germany	202	5.8	France	31	8.8	USA	10	9.8
USA	308	7.5	UK	194	5.5	Colombia	23	6.5	Mexico	9	8.8
France	250	6.1	Iran	183	5.2	Venezuela	18	5.1	Germany	5	4.9
Italy	248	6.1	France	152	4.3	Spain	18	5.1	India	4	3.9
UK	228	5.6	Venezuela	92	2.6	UK	17	4.8	Colombia	3	2.9
Germany	91	2.2	India	91	2.6	Bolivia	13	3.7	France	3	2.9
Sudan	91	2.2	Spain	82	2.3	Peru	11	3.1	Italy	2	2.0
Iran	82	2.0	Colombia	78	2.2	Sudan	7	2.0	Argentina	2	2.0
Netherlands	75	1.8	Switzerland	75	2.1	Italy	5	1.4	Egypt	2	2.0
Tunisia	69	1.7	Israel	74	2.1	Argentina	5	1.4	Iran	2	2.0
Turkey	60	1.5	Tunisia	70	2.0	Germany	5	1.4	Sweden	2	2.0
Greece	59	1.4	Japan	66	1.9	Switzerland	4	1.1	Japan	1	1.0
Portugal	58	1.4	Turkey	63	1.8	India	3	0.8	Lebanon	1	1.0
Canada	57	1.4	Egypt	59	1.7	Japan	3	0.8	Nigeria	1	1.0
Belgium	51	1.2	Canada	58	1.7	Netherlands	3	0.8	Senegal	1	1.0
Switzerland	50	1.2	Italy	57	1.6	Egypt	2	0.6	Spain	1	1.0
Kenya	46	1.1	Pakistan	50	1.4	Tunisia	2	0.6	Burkina Faso	1	1.0
Nepal	45	1.1	Mexico	48	1.4	Israel	2	0.6	Switzerland	1	1.0
Ethiopia	43	1.1	Australia	46	1.3	Denmark	2	0.6	Bolivia	1	1.0
Israel	42	1.0	Argentina	43	1.2	Pakistan	2	0.6	Australia	1	1.0
Venezuela	39	1.0	Kenya	38	1.1	Greece	2	0.6	Tunisia	1	1.0
China	34	0.8	Sudan	34	1.0	Nepal	1	0.3	Iraq	1	1.0
Bangladesh	30	0.7	Saudi Arabia	30	0.9	Madagascar	1	0.3	-	-	-
Denmark	30	0.7	Netherlands	27	0.8	Iran	1	0.3	-	-	-
Egypt	29	0.7	Jordan	22	0.6	Panama	1	0.3	-	-	-
Colombia	26	0.6	Denmark	21	0.6	Canada	1	0.3	-	-	-
Saudi Arabia	19	0.5	Sri Lanka	20	0.6	Saudi Arabia	1	0.3	-	-	-
Pakistan	17	0.4	Belgium	19	0.5	Sri Lanka	1	0.3	-	-	-
**World regions**	**N. of docs**	**%**	**World regions**	**N. of docs**	**%**	**World regions**	**N. of docs**	**%**	**World regions**	**N. of docs**	**%**
Europe	1,554	38.1	Latin America and the Caribbean	987	28.1	Latin America and the Caribbean	205	57.9	Latin America and the Caribbean	62	17.5
Asia	1,022	25.0	Europe	902	25.7	Europe	89	25.1	Europe	14	4.0
Latin America and the Caribbean	674	16.5	North America	629	17.9	North America	34	9.6	North America	10	2.8
North America	365	8.9	Asia	480	13.7	Asia	12	3.4	Asia	7	2.0
North Africa	244	6.0	North Africa	354	10.1	Africa	8	2.3	North Africa	5	1.4
Africa	204	5.0	Africa	113	3.2	North Africa	6	1.7	Africa	3	0.8
Oceania	18	0.4	Oceania	47	1.3	Oceania	0	0.0	Oceania	1	0.3
Total	4.081	100	Total	3,512	100	Total	354	100	Total	102	100

Docs: documents.

### Publication by geographic area

Europe was by far the most productive area in the field of leishmaniasis, responsible for 31.7% of all articles. Latin America and the Caribbean and North America ranked second and third, respectively, with 24.5% and 16.2% each (Table 3). The ranking corrected by population gives the first position to Oceania followed by North America. When normalised by GPD, GNI per capita and HE per capita, the order of prominence was Latin America and the Caribbean (Table 3).

Europe was the most productive in visceral leishmaniasis, followed by Asia. Latin America and the Caribbean ranked first in cutaneous, mucocutaneous and diffuse cutaneous leishmaniasis (Table 4).

**Figure 4:**
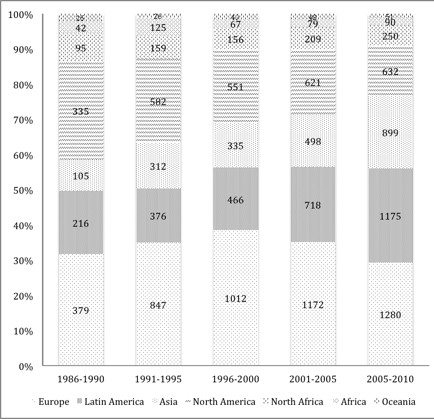
Research output of different world regions in leishmaniasis documents with institutional address of the first author published from 1986 to 2010 per 5-year period.

Every world region increased their absolute production during the study period. Europe had more articles published each 5-year period, but their relative contribution fell during the last one, to 29.2%. Latin America increased its absolute production during the study period and increased its relative contribution from 18.0% in 1986-1990 to 26.8% from 2006-2010. Asia increased its absolute production during the study period, and increased its relative contribution from 8.8% in 1986-1990 to 20.5% in 2006-2010. The relative contribution of North America decreased from 28.0% in 1986-1990 to 14.4% in the 2006-2010 period. The relative contribution of North Africa and the Middle East, Africa and Oceania was similar during all 5-year periods (Figure 4).

### Authorship

Table 5 ranks the 20 most productive authors in each form of the disease. For visceral leishmaniasis, the main author was S. Sundar, an Indian researcher (n = 164 documents), followed by H.W. Murray (n = 91), a North American investigator, and L. Grandoni (n = 87), an Italian scientist. For cutaneous leishmaniasis, the main author was P. Schott (n = 62), a North American investigator, followed by F. Pratlong (n = 55), a researcher from France, and D.L. Sacks (n = 49), a North American scientist. In mucocutaneous leishmaniasis, P.D. Marsden (n = 68), a Brazilian, was the leader, while for diffuse cutaneous leishmaniasis, leading were J.M. Costa, J. Convit and A.C. Saldanha (n = 11 each), among Latin American investigators.

**Table 5:** Twenty most-productive authors ranked according to total number of publications by forms of the disease

**Visceral leishmaniasis**			**Cutaneous leishmaniasis**			**Mucocutaneous leishmaniasis**			**Diffuse cutaneous leishmaniasis**		
**Author**	**N. of docs**	**%**	**Author**	**N. of docs**	**%**	**Author**	**N. of docs**	**%**	**Author**	**N. of docs**	**%**
Sundar. S	164	3.9	Scott. Phillip	62	1.8	Marsden. Philip D	68	19.2	Costa. J M	11	3.2
Murray. Henry W	91	2.2	Pratlong. Francine	55	1.6	Carvalho. Edgar M	30	8.5	Convit. Jacinto	11	3.2
Gradoni. Luigi	87	2.1	Sacks. David L	49	1.4	Lainson. Ralph	28	7.9	Saldanha. Ana Cr	11	3.2
Thakur. Chandreshwar P	76	1.9	Barral. Aldina M	47	1.3	Llanos Cuentas. E A	28	7.9	Barral. Aldina M	10	2.9
Boelaert. Marleen	73	1.8	Carvalho. Edgar M	47	1.3	Cuba. C C	23	6.5	Carvalho. Edgar M	9	2.6
El Hassan. Ahmed M	73	1.8	Dedet. Jean Pierre	47	1.3	Convit. Jacinto	23	6.5	Becker. I	6	1.7
Gramiccia. Marina	62	1.5	Louis. Jacques A	40	1.1	Shaw. Jeffrey J	21	5.9	Ulrich. Marian	6	1.7
Pratlong. Francine	62	1.5	Röllinghoff. Martin	40	1.1	Dedet. Jean Pi	20	5.6	Barral Netto. Manoel	5	1.4
Alvar. Jorge	60	1.5	Locksley. Richard M	40	1.1	Costa. J M	19	5.4	Bittencourt. Achiléa L	5	1.4
Marty. Pierre	56	1.4	Mayrink. Wilson	38	1.1	Barreto. A C	19	5.4	Tapia. Felix J	5	1.4
Dedet. Jean P	55	1.3	Handman. Emanuela	37	1.1	Netto. Eduardo M	19	5.4	Galvão. C E	4	1.2
Kaye. Paul M	53	1.3	Launois. Pascal	35	1.0	Saravia. Nancy G	18	5.1	Cáceres Dittmar. G	4	1.2
Kager. Piet A	52	1.3	Barral Netto. Manoel	35	1.0	Pirmez. Claude	17	4.8	Pratlong. Francine	4	1.2
Reed. Steven G	50	1.2	El Hassan. Ahmed M	35	1.0	Grimaldi Júnior. Gabriel	16	4.5	Pacheco de Almeida. Roque	3	0.9
Rijal. Suman	50	1.2	Titus. Richard G	34	1.0	Barral. Aldina Maria P	16	4.5	Dedet. Jean P	3	0.9
Badaró. Roberto	47	1.2	Llanos Cuentas. E A	32	0.9	Lessa. Hélio Andrade	16	4.5	Meyer Fernandes. José R	3	0.9
Carvalho. Edgar M	46	1.1	Khamesipour. Ali	30	0.9	Furtado. T A	14	4.0	Machado. Paulo R L	3	0.9
Davidson. Robert N	41	1.0	Shaw. Jeffrey Jon	30	0.9	Sampaio. Raimunda N R	13	3.7	Pérez Montfort. Ruy	3	0.9
Croft. Simon L	41	1.0	Ramesh. Venkatesh	29	0.8	Mayrink. Wilson	13	3.7	Guimarães. Luis H	3	0.9
Pearson. Richard D	41	1.0	Moll. Heidrun	28	0.8	Christensen. H A	13	3.7	Costa. Jackson Maurício L	3	0.9

Docs: documents.

## Discussion

This study has shown an increase in the number of publications on leishmaniasis over the 1945-2010 period, which seems to be more pronounced than that observed in other neglected tropical diseases, such as American trypanosomiasis or leprosy [17,18] and global tropical medicine [12-14]. Moreover, scientific publications on leprosy have experienced a reduced trend since the turn of the century [17]. This is probably related to different causes. First, the increase in estimating the prevalence of leishmaniasis seen in recent years [1]. Secondly, greater social awareness, including by the pharmaceutical industry and philanthropic world that has opened to these diseases, including the Bill & Melinda Gates Foundation and other non-governmental organizations. Thirdly, we do not doubt the important steps taken by the World Health Organization (WHO) for inclusion in its health agenda an initiative to control the disease in endemic countries [2]. Fourthly, the therapeutic discoveries of drugs over the past 15 years (amphotericine B liposomic, miltefosine and paromomycin), or diagnosis procedures attract much attention on leishmaniasis [2,4,24,25]. Therefore, the continuous interest in the field and the incorporation of new journals in PubMed may have contributed to this linear increase. In this sense, two outstanding open access journals devoted to the study of Tropical Diseases have been launched in recent years: *PLoS Neglected Tropical Diseases*, started in 2007; and *Parasites & Vectors*, established in 2008. Even though they do not appear in long-term bibliometric analyses, they are playing a major role in the area with a strong research activity.

Although the main language of leishmaniasis research output is English (82.8%), this language is less common than other bibliometric studies based on PubMed, where 85-90% of its documents are in English [8,9]. The other more important languages were French and Portuguese. Leishmaniasis is endemic in North Africa, France and Brazil [1], which might explain the prevalence of these languages with respect to others. For instance, the geographic distribution of the disease is important with the publication language about the disease [26]. For this, reviewing the journals in the original language of geographical distribution of the diseases is interesting.

*Journal articles* were the most commonly retrieved document type (approximately 90%), similar to other bibliometric studies on NTDs and non-NTDs [8-10,17,18]. Although controlled trials offer the best evidence for medical intervention efficacy [27], in this study they represented only 0.3% of the documents, a figure lower than in other fields [8,9] and similar to other NTDs [18].

Nucleus journals usually contain articles with the highest impact in the area and thus, subscriptions to such journals in indexing and abstracting services would be justified scientifically [10,28]. Most top journals publishing on leishmaniasis were from the *parasitology*, *immunology*, and *tropical medicine* subject categories. The top journal was *Transactions of the Royal Society of Tropical Medicine and Hygiene* from the UK, while the *American Journal of Tropical Medicine and Hygiene* from the USA was second. Both included the *public, environmental and occupational health* and *tropical medicine* subject categories. The fourth and seventh journals were *Memórias do Instituto Oswaldo Cruz* and *Annals of Tropical Medicine and Parasitology* including the *parasitology* and *tropical medicine* subject categories. The third and fifth journals were *Molecular and Biochemical Parasitology* and *Experimental Parasitology*; both journals publish basic aspects of parasitology. The sixth and eighth journals were the *Journal of Immunology* and *Infection and Immunology*, respectively, both related to immunology.

USA was the leading country in publication output on leishmaniasis, like that which has also been described in other biomedical fields [8-10], although the number of leishmaniasis cases there is less than in South America. Brazil, a country with a high prevalence of leishmaniasis, led scientific production on leishmaniasis in Latin America. This can be attributed to the number of researchers and development of the country’s scientific system, which has become the principal scientific reference for South America [20,29]. India, a country with a high prevalence of leishmaniasis, mainly in the state of Bihar [1,2,25,30], was the third country, and it led scientific production on leishmaniasis in Asia.

Leishmaniasis research in small countries, after adjusting for population, was led by Israel and Switzerland. Swiss publications came mainly from the WHO, especially from the Programmes of *Prevention and Control of Leishmaniasis* and *Drugs for Neglected Diseases initiative*[1,2]. Although institutions of the United Nations are not attributed to any country, the WHO is physical located in Geneva, Switzerland. The leading countries after adjusting for GDP were low- or middle-income countries with a higher prevalence of leishmaniasis, like Nepal, Tunisia, Kenya, Ethiopia and Sudan. When adjusting economic and demographic aspects (GNI per capita), the leading countries were low- and middle-income countries with a higher prevalence of leishmaniasis and overpopulated, like India, Ethiopia, Pakistan and Brazil [24,30,31].

In visceral leishmaniasis, the leading countries were India and Brazil, both with high prevalence. Spain was the third country, probably influenced by the association of visceral leishmaniasis and HIV infection [32]. In cutaneous leishmaniasis, Brazil, USA and Germany topped the rankings.

Europe was the world’s leading area in scientific production on leishmaniasis. Although the disease load in Europe constitutes less than 0.2% of global cases of leishmaniasis, there are people with *Leishmania* infections living there, especially along the Mediterranean Coast [1]. Europe has a long tradition of agencies and institutions implementing research and health programmes in tropical medicine and parasitology [33-35], in addition to networks of scientists operating in these countries with other countries where leishmaniasis is endemic. Latin America was the world’s second-leading area in scientific production on leishmaniasis. Its relative contribution increased to 26.8% in the last 5-year study period. This is because of a high prevalence of this disease on this continent, the long lasting interest in this field in the Latin American countries, especially Brazil [19,24,29,35-37], and the incorporation of new Latin American journals in PubMed. North America was the world’s third area. However, its relative contribution decreased to 14.4% in the 2006-2010 period. A decreased contribution from North America also occurred in other biomedical fields such as tuberculosis [8]. The absolute and relative contribution from Asia increased in our study from 105 (8.8%) in 1986-1990 to 899 (20.5%) in the 2006-2010 period. This increasing Asian contribution has been seen in other scientific fields because of their improving research, including many leishmaniasis clinical trials conducted on the Indian subcontinent, and increasing incorporation of new Asians journals in PubMed [38]. The relative contribution from North Africa and the Middle East, Africa and Oceania was similar during all 5-year periods.

Europe and North America are at the vanguard of scientific excellence and development, and should increase their collaboration with scientific publications in developing countries, especially from North Africa and the Middle East and Africa in the field of leishmaniasis. Europe was the world leader in visceral leishmaniasis, followed by Asia. However, Latin America and the Caribbean ranked first in cutaneous, mucocutaneous and diffuse cutaneous leishmaniasis.

PubMed and the *Science Citation Index* were found to be the most suitable databases for searching and retrieving references for bibliometric studies [8,21]. We used the PubMed database because it is easily accessible and widely used, it uses a controlled vocabulary for indexing and recovering documents [8,9,39], and the index journal in Medline has a certain criteria for quality [16,40]. However, the method we used may have several limitations that have been explained in other publications [18]. For example, the database mainly includes journals published in English, and journals in other languages are less likely to be found on PubMed. However, this database has more non-English journals than the Web of Science database [41]. Another limitation is that in PubMed, only the address of the first author appears in the journal articles, whereas in *letters* and *editorials*, the address field is not recorded, and the address has only been included since 1986 and systematically ever since. For instance, estimating the quantity of articles resulting from multinational collaborations was not possible. This may cause some problems when estimating research productivity from developing countries that work in collaboration with scientists from a developed country. Even though the bibliometric methodology used may present some limitations and the results could, in some way, be biased [9,40], we believe that this study represents a useful tool for scientists and public health policy makers in planning and organizing research in the field of leishmaniasis. We should emphasize that other authors should employ the method we used to find research production, so that our results may be comparable to others in the future.

## Conclusion

In conclusion, we have found an increase in the number of publications in the field of leishmaniasis disease. Authors affiliated to institutions in USA and Brazil led scientific production on leishmaniasis research. Efforts should be made to help developing countries with the highest prevalence of leishmaniasis develop scientific research networks (collaborative platforms) with North American and/or European countries in order to increase research with interdisciplinary teams [42].

## Competing interests

The authors declare that they have no competing interests.

## Authors’ contributions

JMR and GGA participated in the design and co-ordination of the study, performed the analysis, and drafted and prepared the paper. JMR, GGA, and MBP participated in the analysis. All authors read and approved the final document.
